# Supplementary material for: A gated hydrophobic funnel within BAX binds bioactive lipids to potentiate pro-apoptotic function
Source: Nat Commun. 2026 Feb 25;17:3180. doi: 10.1038/s41467-026-69836-9 (PMC13046825; doi:10.1038/s41467-026-69836-9)
Supplement: Supplementary file 1 — Supplementary Information [file 41467_2026_69836_MOESM1_ESM.pdf]

A gated hydrophobic funnel within BAX binds bioactive lipids to potentiate pro-apoptotic function

Jesse D. Gelles, Yiyang Chen, Mark P. A. Luna-Vargas, Ariele Viacava Follis, Md Abdullah Al Noman, Md Kabir, Stella G. Bayiokos, Jarvier N. Mohammed, Tara M. Sebastian, Ngoc Dung Pham, Yi Shi, Jian Jin, Richard W. Kriwacki, and Jerry Edward Chipuk

SUPPLEMENTARY INFORMATION

Supplementary Figures and Figure Legends 1–10

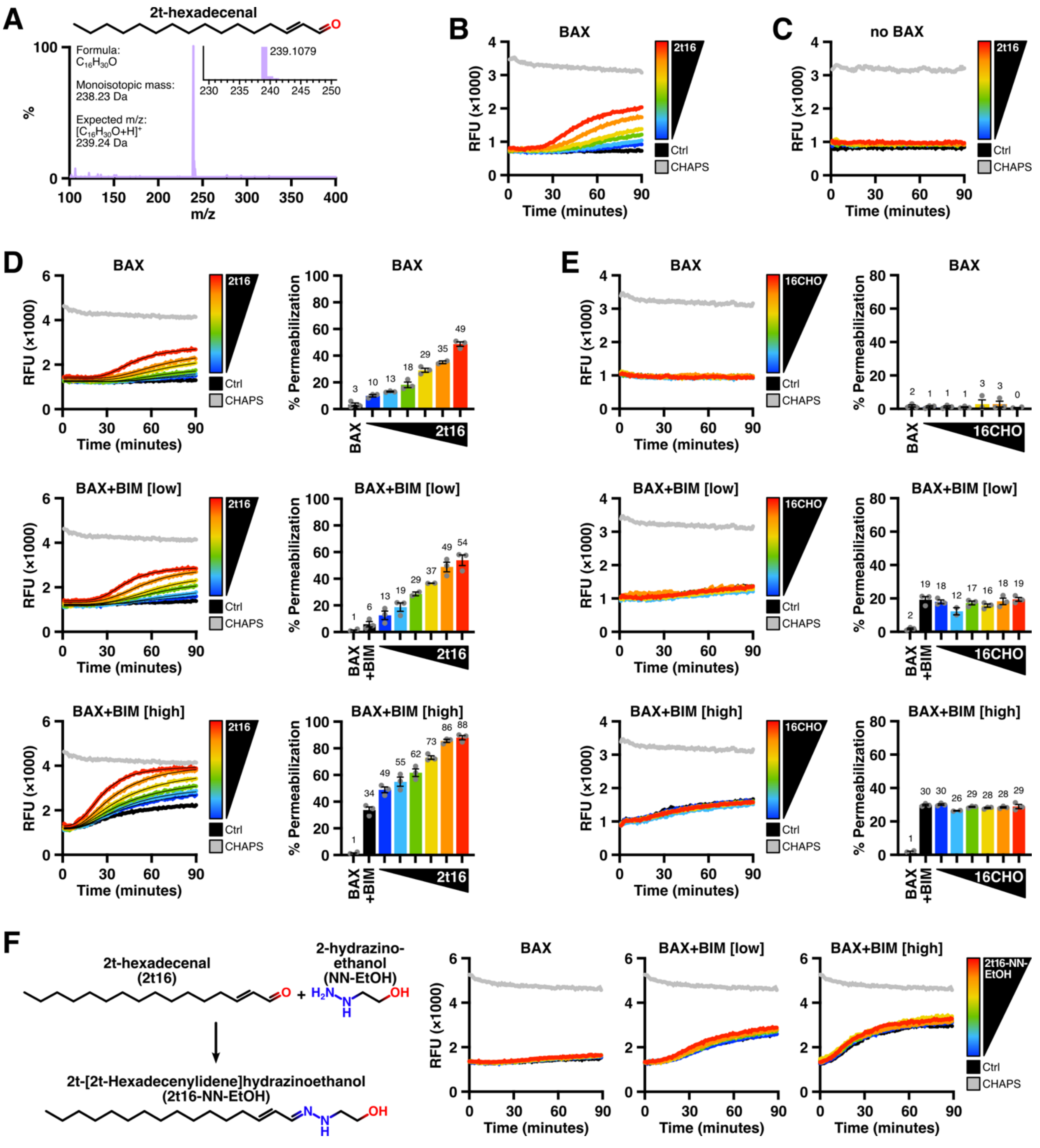

Supplementary Figure 1

Supplementary Figure 1: 2t-hexadecenal promotes BAX functionalization (Related to Figure 1)

(A) Structure and mass of 2t-hexadecenal was validated by TOF mass spectrometry to confirm reagent purity and integrity.

(B–F) LUV permeabilization studies with recombinant BAX protein treated as indicated and measured at regular intervals for changes in fluorescence as fluorophores are released from compromised liposomes. Left panels: kinetic fluorescence data; right panels: endpoint data normalized to LUV fluorescence and maximal signal generated by LUVs solubilized with CHAPS detergent (grey data). Data are shown as the mean of technical replicates and error bars report SEM.

(B) BAX protein (120 nM) was combined with DMSO vehicle or 2t16 (6.5–50 µM) followed by addition of LUVs and measured by fluorescence spectroscopy.

(C) LUVs treated with 2t16 (16.5–50 µM) in the absence of BAX to confirm no membrane destabilization by 2t16.

(D) Data summarized by Figure **1E**. LUVs permeabilized by BAX (120 nM) treated with 2t16 (6.5–50 µM) ± BIM-BH3 peptide (0.5, 2.5 µM). Nonlinear regression of kinetic data is shown and reported in Figure **1F**. Bliss synergy scores were determined from endpoint data and reported in Figure **1G**.

(E) Data summarized by Figure **1H**. LUVs permeabilized by BAX (160 nM) treated with 16CHO (6.5–50 µM) ± BIM-BH3 peptide (0.5, 2.5 µM). Bliss synergy scores were determined from endpoint data and reported in Figure **1I**.

(F) Left: Structures and reaction of 2t-hexadecenal with 2-hydrazinoethanol to modify the aldehyde functional group (2t16-NN-EtOH). Right: Data summarized by Figure **1J**. LUVs permeabilized by BAX (160 nM) treated with 2t16-NN-EtOH (6.5–50 µM) ± BIM-BH3 peptide (0.5, 2.5 µM). Bliss synergy scores were determined from endpoint data and reported in Figure **1K**.

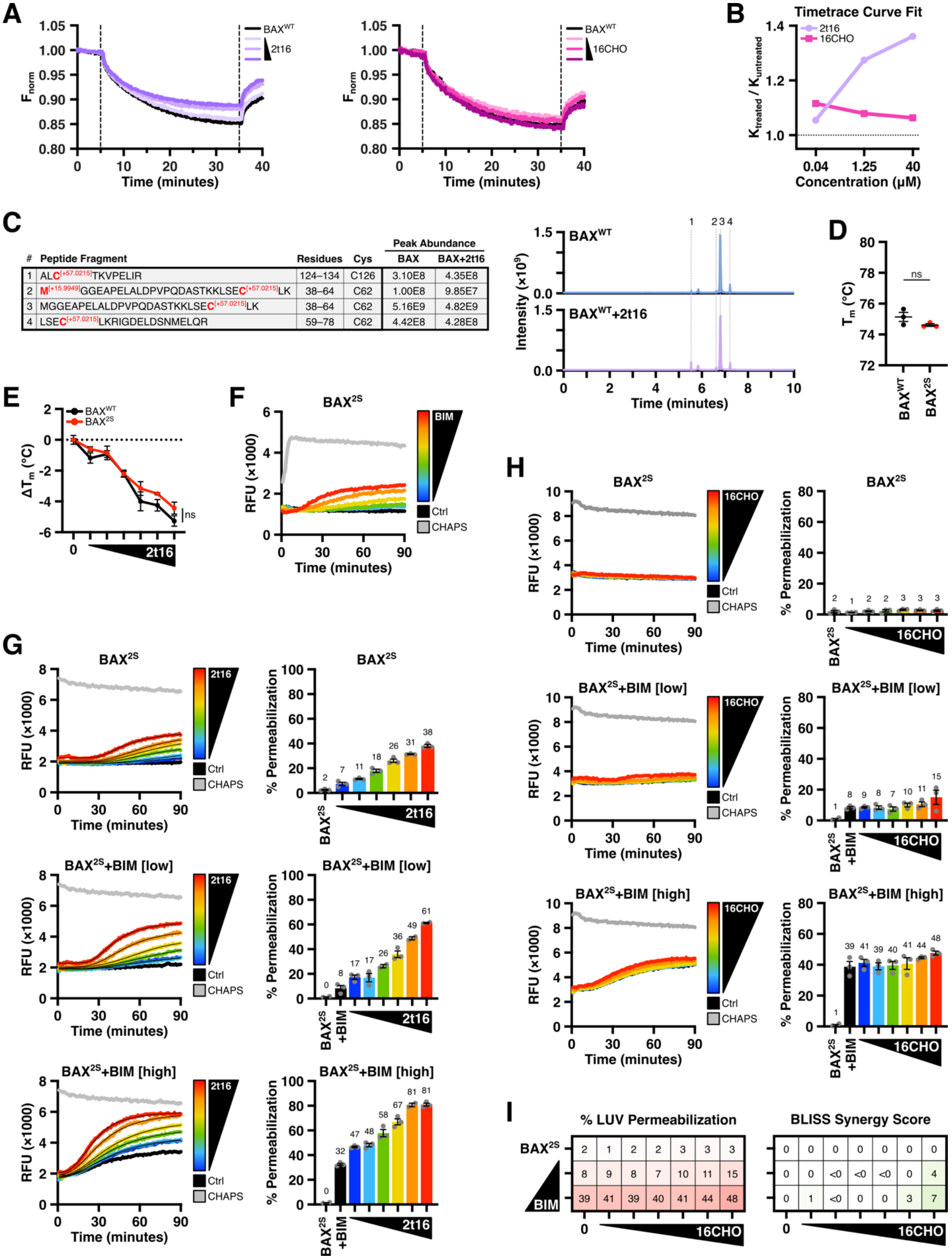

Supplementary Figure 2

Supplementary Figure 2: BAX activation by 2t-hexadecenal does not require cysteine residues (Related to Figure 2)

(**A**) Alexa Fluor 647-labeled recombinant BAX^{WT} (1 nM) was incubated with CHAPS (0.002%) to inhibit oligomerization, treated as indicated, and subjected to MST. Timetrace thermal shift curves of BAX^{WT} titrated with 2t16 or 16CHO (0.04, 1.25, 40 µM) from a representative experiment shown.

(B) Data from **A** was fit using a one-step exponential function and compared using the calculated decay constants (K) normalized to the untreated sample.

(C) LC-MS of recombinant BAX^{WT} alone or incubated with 2t16. Samples were then alkylated with iodoacetamide to identify unmodified cysteine residues and trypsin digested for analysis. Four cysteine-containing peptide fragments were detected. Values denote peptide abundance, calculated as AUC for each peak.

(D–E) The melting temperature of BAX^{WT} and BAX^{2S} ± 2t16 (6.5–50 µM) was measured by thermal shift assay using SYPRO orange and compared using three replicate samples. Individual values, the mean, and SEM are shown. Statistical significance was determined by paired t test; ns, not significant ($P = 0.144$). Data shown are representative of replicated experiments.

(F–H) LUV permeabilization studies with recombinant BAX^{2S} protein treated as indicated and repeatedly measured for changes in fluorescence as fluorophores are released from compromised liposomes. Left panels: kinetic fluorescence data; right panels: endpoint data normalized to LUV fluorescence and maximal signal generated by LUVs solubilized with CHAPS detergent (grey data). Data are shown as the mean of technical replicates and error bars report SEM.

(F) BAX^{2S} (100 nM) was activated by BIM-BH3 peptide (0.13–2 µM) and added to LUVs to confirm sensitivity to activation and pore formation.

(G) Data summarized by Figure **2I**. LUVs permeabilized by BAX^{2S} (100 nM) treated with 2t16 (6.5–50 µM) ± BIM-BH3 peptide (0.5, 2.5 µM). Nonlinear regression of kinetic data is shown as overlayed black curves. Bliss synergy scores were determined from endpoint data.

(H) LUV permeabilization studies as in **G** with BAX^{2S} (100 nM) and hexadecanal (6.5–50 µM) ± BIM-BH3 peptide (0.5, 2.5 µM).

(I) Endpoint data (left panel) and Bliss synergy scores (right panel) from data in **H**.

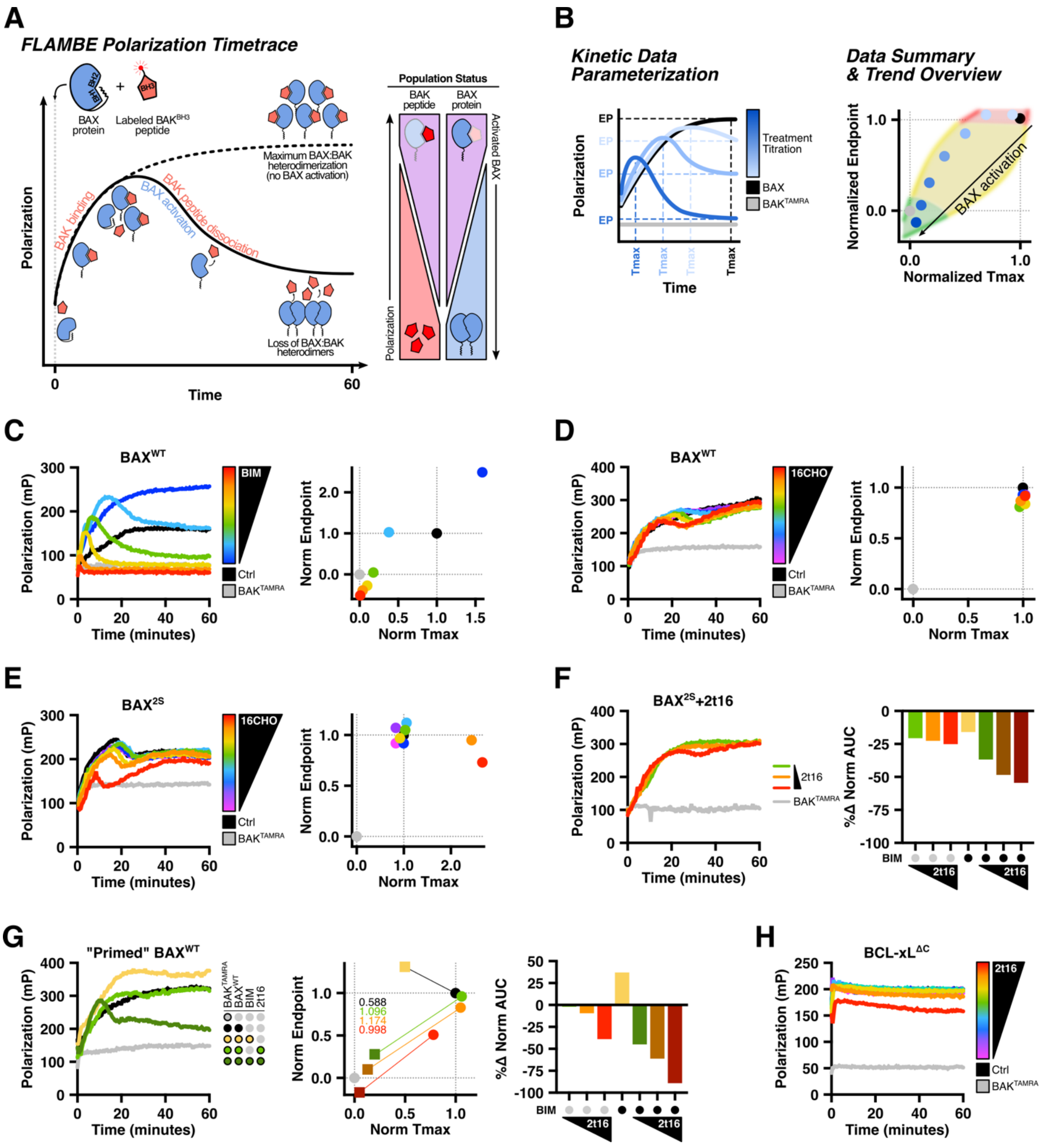

Supplementary Figure 3

Supplementary Figure 3: An assay for detecting BAX early-activation steps reveals that 2t-hexadecenal cooperates with BIM-mediated triggering (Related to Figure 3)

(A) Illustration of BAX and BAK-BH3 interactions within FLAMBE. The reporter is a fluorescently-labeled BAK-BH3 peptide that exhibits changes in Polarization measurements as it is bound by BAX. Over time, the BAX population binds BAK-BH3 peptides resulting in increased Polarization (dotted line). In conditions activating BAX, activation-induced intramolecular rearrangements induce dissociation of the BAK-BH3 peptide with a concomitant decrease in Polarization over time as the population of unbound BAK-BH3 increases (solid line).

(B) Illustration of FLAMBE data parameterization and analysis. Left: Kinetic Polarization data for a treatment inducing BAX activation and exhibiting BAK^{TAMRA} dissociation. Kinetic data is parameterized using endpoint Polarization (EP) and time-to-maximum peak (Tmax) for each condition. Right: Parameterized data are normalized to controls and visualized as a two-dimensional plot. Generally, data approaches lower-left region with potency of BAX activation (yellow and green regions). Conditions plotted with EP > 1, form stable non-activating complexes with the $BAX{:}BAK^{TAMRA}$ heterodimer (red region).

(C) BAX^{WT} (60 nM) was treated with BIM-BH3 peptide (0.25–2 µM) and subjected to FLAMBE to visualize dose-dependent activation-induced dissociation of BAK^{TAMRA}. BIM-BH3 at a low concentration (0.25 µM, dark blue) demonstrated a stable, non-activating interaction with the $BAX{:}BAK^{TAMRA}$ complex and exhibited increased Polarization.

(D–E) BAX^{WT} (60 nM) and BAX^{2S} (60 nM) was treated with 16CHO (2–50 µM), combined with BAK^{TAMRA} (50 nM), and subjected to FLAMBE.

(F) Left: BAX^{2S} (60 nM) was treated with three non-activating concentrations of 2t16 (green: 4.5 µM; orange: 6.5 µM; red: 10 µM). Parameterization of this data is included in **Figure 3D**. Right: AUC calculated for each condition was normalized to the BAX and BAK^{TAMRA} controls and reported as a percent change from the vehicle-treated BAX condition.

(G) Left: BAX^{WT} (60 nM) was combined with a non-activating concentrations of BIM-BH3 peptide (0.15 µM) and 2t16 (4.5 µM), followed by BAK^{TAMRA} (50 nM), and subjected to FLAMBE. Middle: Parameterized FLAMBE data including three concentrations of 2t16 (green: 4.5 µM; orange: 6.5 µM; red: 10 µM) in the absence or presence of BIM-BH3 (circle and square datapoints, respectively). Annotations report the magnitude of shift between data with and without BIM-BH3. Right: AUC calculated for each condition was normalized to the BAX and BAK^{TAMRA} controls and reported as a percent change from the vehicle-treated BAX condition.

(H) Fluorescence polarization competition assay with recombinant BCL-$xL^{\Delta C}$ protein treated with 2t16 (2–50 µM) and combined with BAK^{TAMRA}.

Panels **A**–**B** are adapted from a previously published version of this diagram (Gelles et al., 2022; 10.1016/j.crmeth.2022.100174)

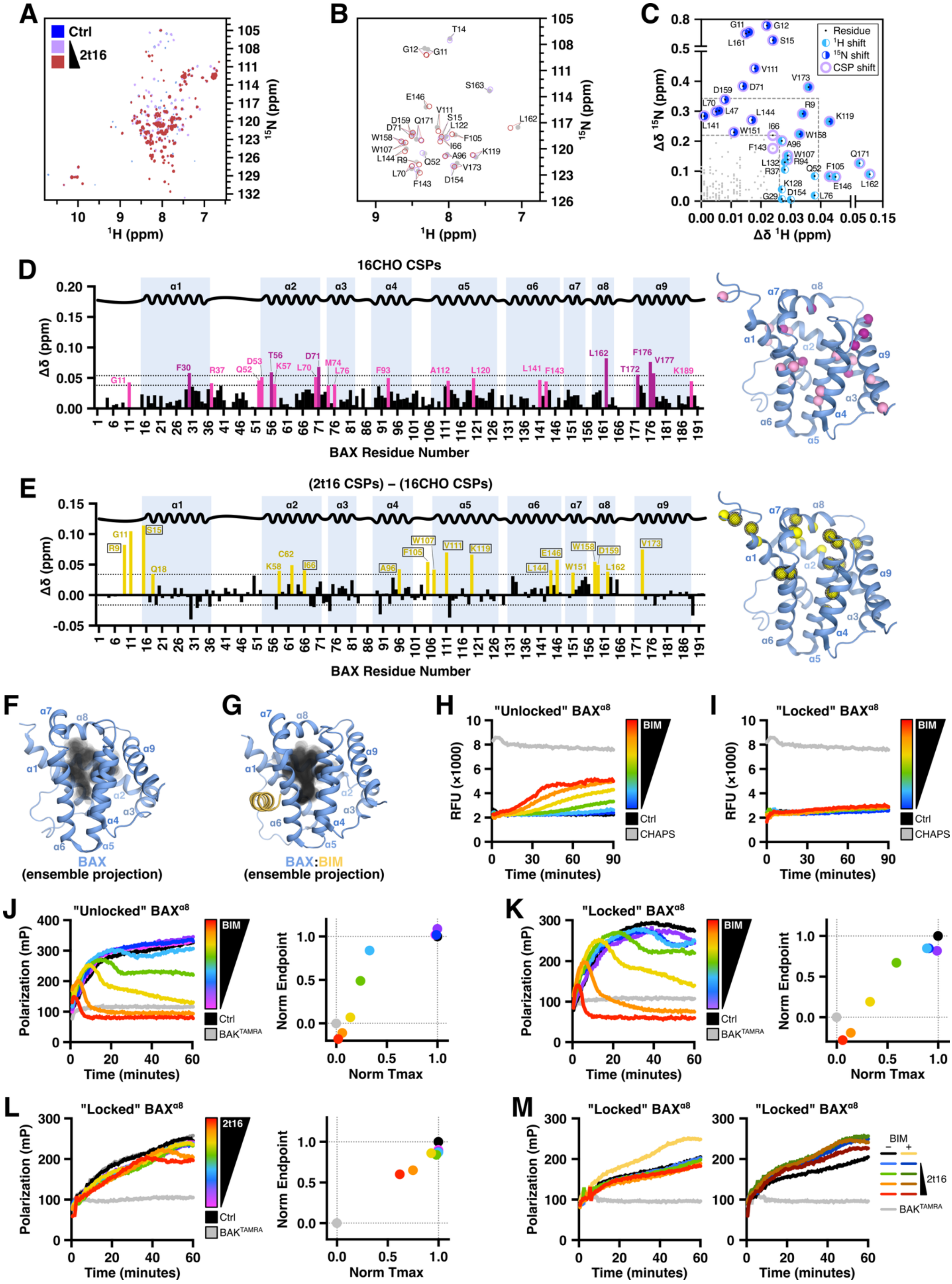

Supplementary Figure 4

Supplementary Figure 4: Structural, biophysical, and functional approaches identify α8 mobilization as necessary for 2t-hexadecenal function (Related to Figure 4)

(A) ^{1}H-^{15}N HSQC NMR spectra of ^{15}N-labeled BAX^{WT} (40 µM) treated with vehicle or 2t16 (50, 150 µM).

(B) Plot depicting peak shifts of BAX residues exhibiting a significant CSP value in response to incubation with 2t16.

(C) Plot of individual values of ^{1}H or ^{15}N shifts. Residues exhibiting a shift greater than 1 standard deviations above the average are highlighted (light blue left semicircle for ^{1}H dimension, dark blue right semicircle for ^{15}N dimensions, and purple for combined CSP determination).

(D) Chemical shift perturbations (CSPs) observed in ^{15}N-labeled BAX^{WT} incubated with 16CHO (150 µM). Residues exhibiting a shift greater than 1 or 2 standard deviations above the average (dotted lines) are highlighted and indicated on the BAX structure (PDB: 1F16). The absence of a bar indicates no chemical shift difference, the presence of a proline, or the residue that could not be definitively assigned.

(E) Chemical shift perturbations (CSPs) observed in ^{15}N-labeled BAX incubated with 2t16 (Figure **4A**) were subtracted by CSPs observed with 16CHO (Figure **S4C**) and plotted as a function of BAX residues. Residues exhibiting a shift greater than the 1 standard deviation above the average (dotted line) are colored yellow and indicated on the BAX structure (PDB: 1F16). Residues uniquely exhibiting significance in the 2t16 treatment and not 16CHO are highlighted with a black outline. The absence of a bar indicates no chemical shift difference, the presence of a proline, or the residue that could not be definitively assigned.

(F) Cartoon visualization of BAX (PDB: 1F16) and the hydrophobic cavity in the BAX core determined for each of the 20 ensemble states and overlayed to assess general morphology. Cavity determination and visualization was performed with PyMOL using a cavity radius of 3 and a cavity cutoff of -5.5.

(G) Cartoon visualization as in **F** for BIM-bound, triggered BAX (PDB: 2K7W).

(H) LUVs permeabilized by reduced $BAX^{\alpha 8}$ (220 nM) treated with BIM-BH3 peptide (0.2–6.5 µM). Grey data report LUVs solubilized with 1% CHAPS to measure maximal signal. Data shown are the mean of technical replicates.

(I) Same as in **H** with oxidized $BAX^{\alpha 8}$ (220 nM) to induce a disulfide tether immobilizing α8.

(J–M) $BAX^{\alpha 8}$ was treated as indicated, combined with a TAMRA-labeled BAK-BH3 peptide (BAK^{TAMRA}), and immediately subjected to FLAMBE analysis. Left panels: kinetic Polarization data; right panels: two-dimensional plot of parameterized FLAMBE data comparing Tmax and endpoint Polarization metrics normalized to BAK^{TAMRA} (grey data) and the vehicle-treated BAX control (black data).

(J) Reduced $BAX^{\alpha 8}$ (75 nM) was treated with BIM-BH3 (0.06–1 µM), combined with BAK^{TAMRA} (50 nM), and subjected to FLAMBE.

(K) Same as in **J** with oxidized $BAX^{\alpha 8}$ (250 nM) to immobilize α8.

(L) Oxidized $BAX^{\alpha 8}$ (250 nM) was treated with 2t16 (3–50 µM), combined with BAK^{TAMRA} (50 nM), and subjected to FLAMBE.

(M) $BAX^{\alpha 8}$ (225 nM) was treated with four non-activating concentrations of 2t16 (blue: 3 µM; green: 4.5 µM; orange: 6.5 µM; red: 10 µM) in the absence or presence of BIM-BH3, followed by BAK^{TAMRA} (50 nM), and subjected to FLAMBE. Parameterization of this data is included in **Figure 4I**.

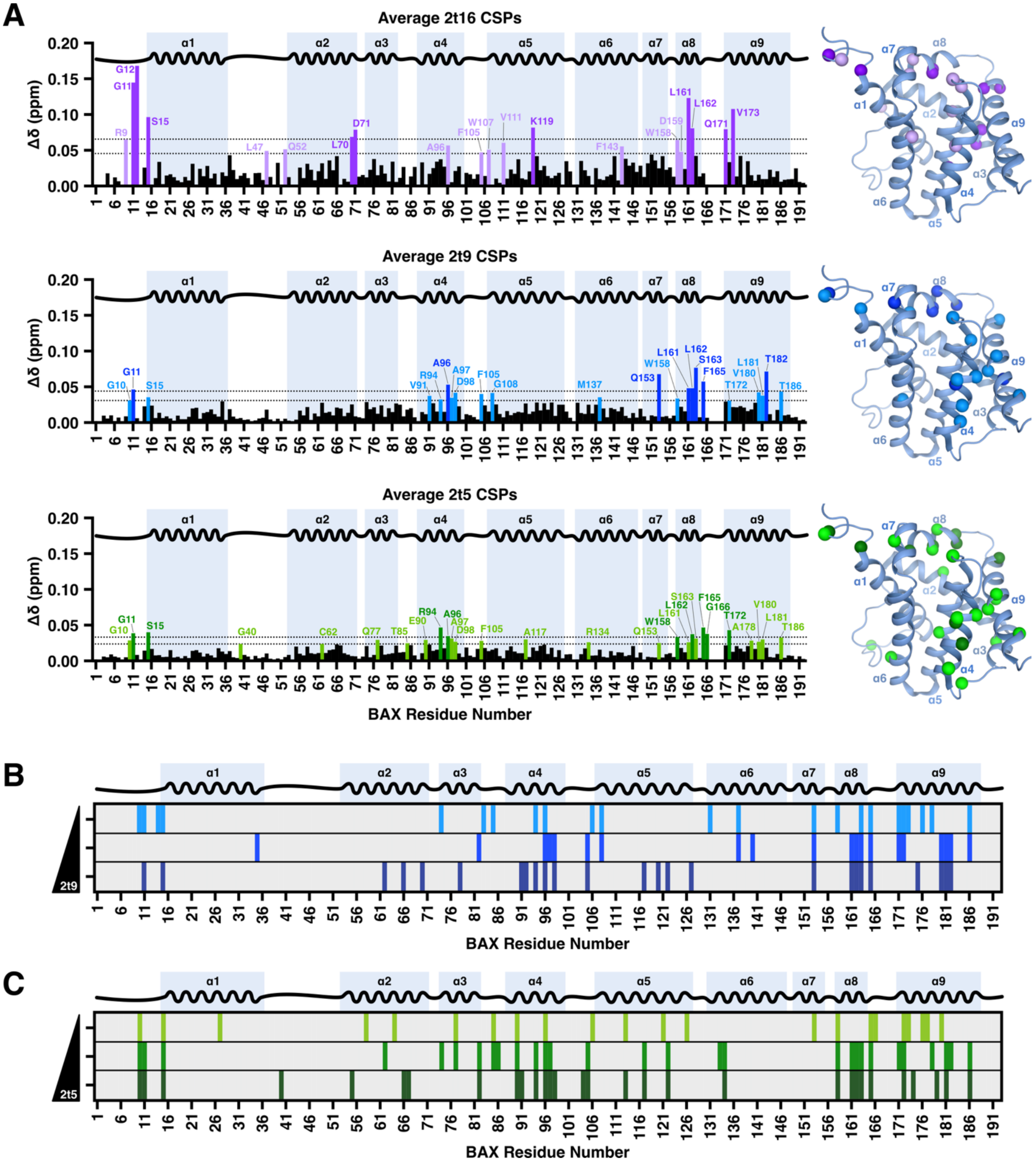

Supplementary Figure 5

Supplementary Figure 5: ^{1}H-^{15}N HSQC perturbations indicate that short-chain 2t-alkenals exhibit CSPs outside of the BAF region (Related to Figure 5)

(A) Chemical shift perturbations (CSPs) observed in ^{15}N-labeled BAX incubated with either 2t16 (50, 150 µM), 2t9 (0.3, 0.9, 2.7 mM), or 2t5 (0.3, 0.9, 2.7 mM) averaged across concentrations. Residues exhibiting a shift greater than 1 or 2 standard deviations above the average (dotted lines) are indicated in light and dark colors, respectively, and indicated on the BAX structure (PDB: 1F16). The absence of a bar indicates no chemical shift difference, the presence of a proline, or the residue that could not be definitively assigned.

(B–C) Residues exhibiting significant CSPs for each concentration (0.3, 0.9, 2.7 mM) of 2t9 or 2t5. Highlighted residues exhibited shifts greater than 1 standard deviation above the average of measurable shifts.

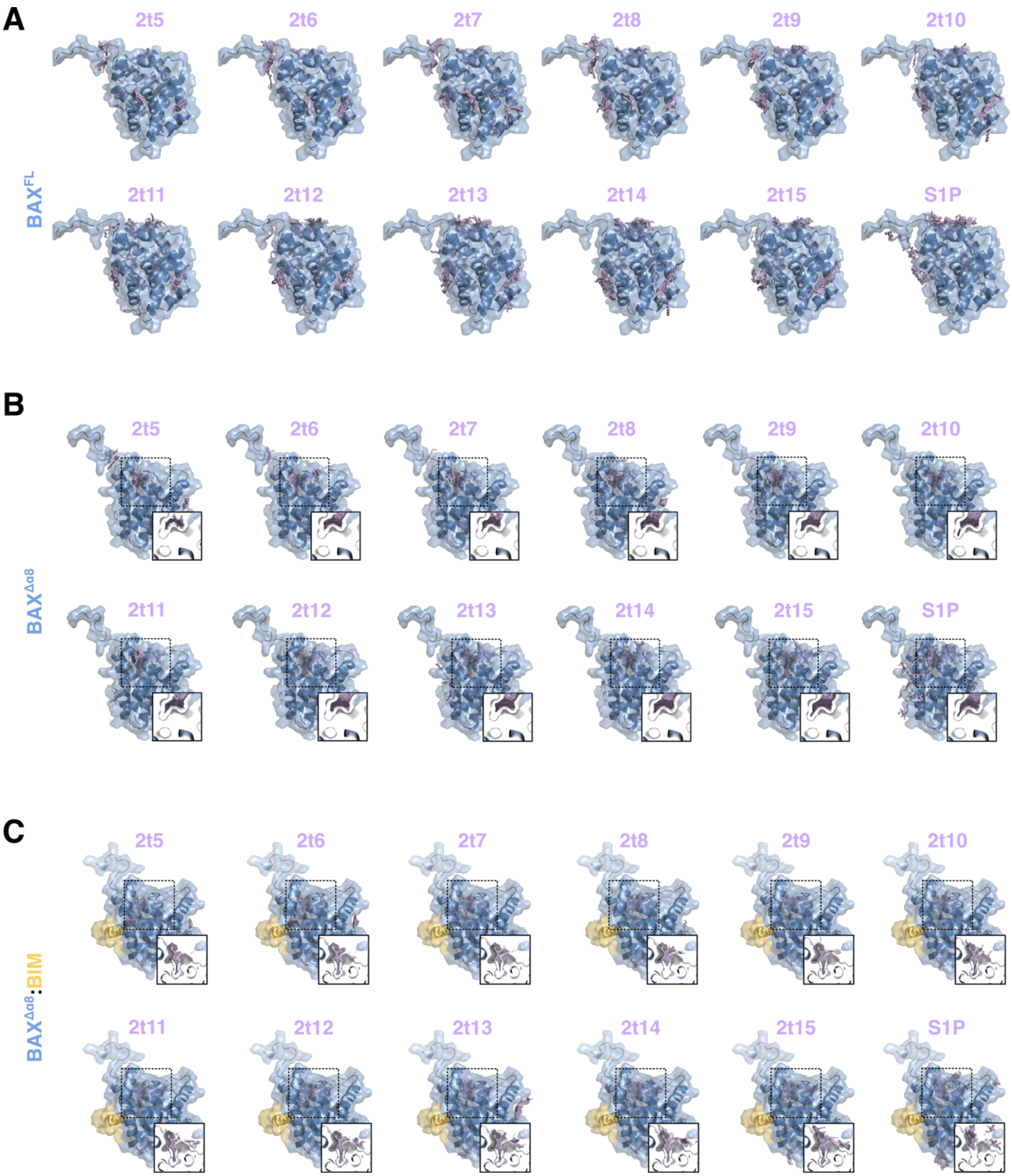

Supplementary Figure 6

Supplementary Figure 6: In silico docking determinations suggest long-chain 2t-alkenals exhibit increased specificity for the BAF (Related to Figure 5)

(A–C) Unconstrained *in silico* rigid docking of 2t-alkenals or S1P against BAX using the SwissDock web service. Insets display cross-section of ligands docking within the BAF. Models visualized with PyMOL.

(A) Visualization of results for each 2t-alkenal or S1P docking on an unmodified structure of BAX (PDB: 1F16, BAX^{FL}). Quantification of results summarized in Figure **5B**.

(B) As in **A** with ligands docking on a structure of BAX with alpha helix 8 removed (PDB: 1F16, Δ157–163; $BAX^{\Delta\alpha8}$). Quantification of results summarized in Figure **5B**.

(C) As in **B** with ligands docking on a structure of BAX bound to a BIM-BH3 peptide with alpha helix 8 removed (PDB: 2K7W, Δ157–163; BAX:BIM).

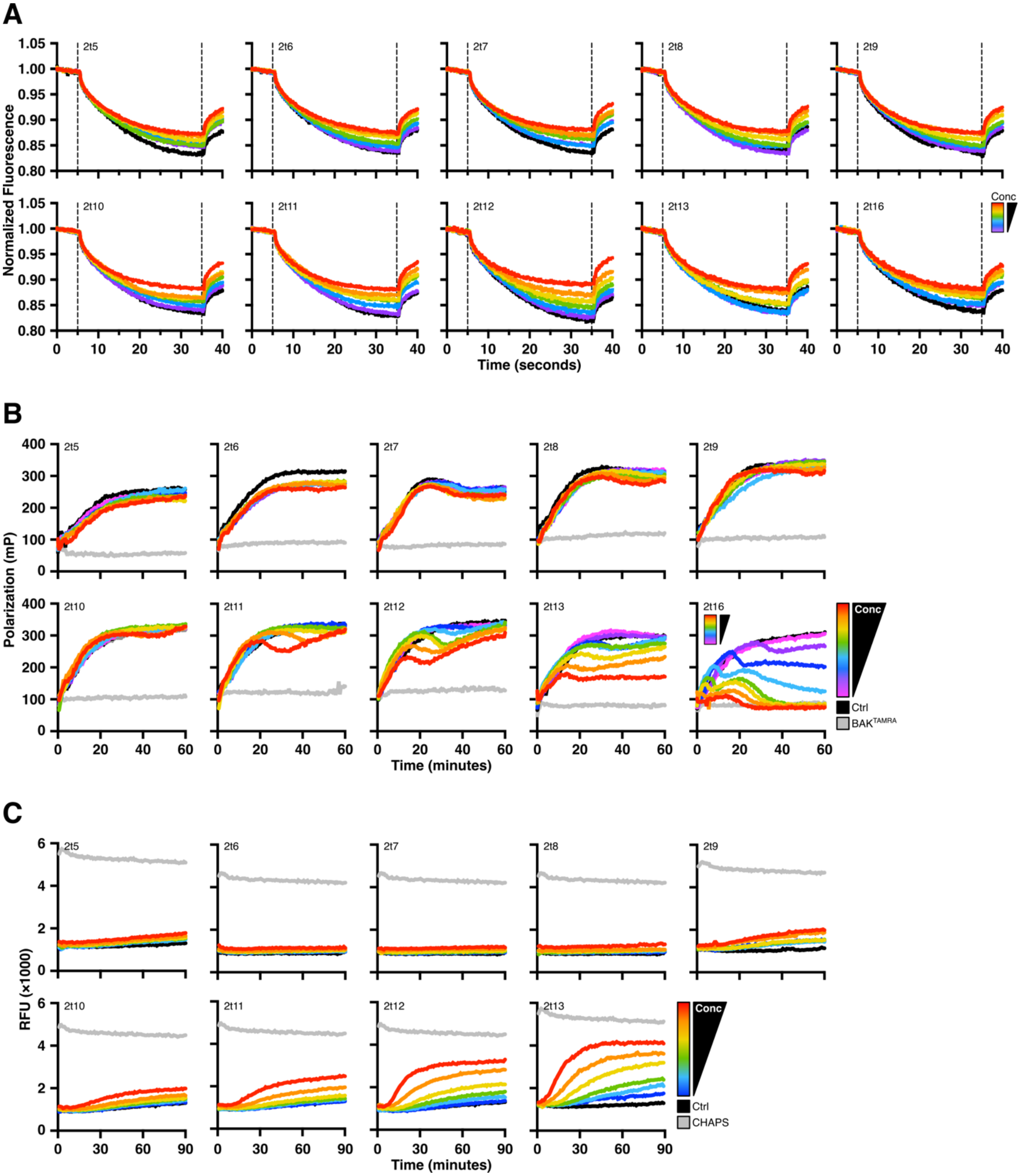

Supplementary Figure 7

Supplementary Figure 7: Long-chain 2t-alkenals are capable of BAX activation (Related to Figure 5)

(A) Alexa Fluor 647-labeled recombinant BAX^{WT} (1 nM) was incubated with CHAPS (0.002%) to inhibit oligomerization, treated with the indicated 2t-alkenals (0.16–5 µM), and subjected to MST. Timetrace data are shown as the mean of replicates. Thermophoresis metrics for each 2t-alkenal are summarized in Figure **5C**.

(B) BAX^{2S} (60 nM) was treated with the indicated 2t-alkenal (3–50 µM), combined with BAK^{TAMRA} (50 nM), and subjected to FLAMBE. Data are shown as the mean of replicates. Parameterized data reporting EP and Tmax for each 2t-alkenal are provided in Figures **5D–E**.

(C) LUVs permeabilized by BAX^{2S} (100 nM) treated with the indicated 2t-alkenal (6.5–50 µM). Data are shown as the mean of replicates. Normalized endpoint permeabilization data summarized in Figure **5F**.

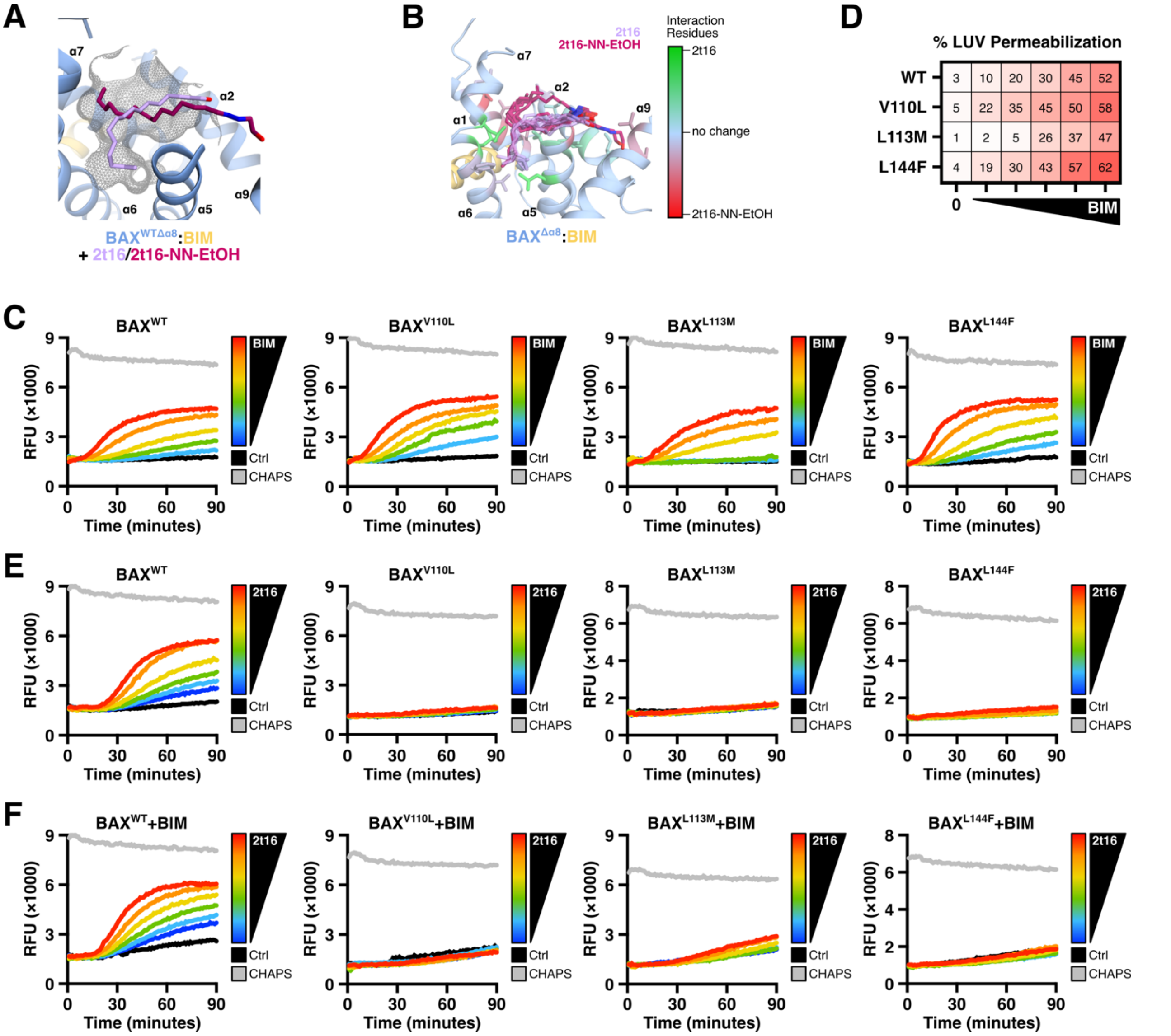

Supplementary Figure 8

Supplementary Figure 8: BAF mutations ablate 2t-hexadecenal mediated membrane permeabilization (Related to Figure 6)

(A) Molecular docking and modeling of $BAX^{\Delta\alpha8}$:BIM (PDB: 2K7W, Δ157–163) with 2t16-NN-EtOH using Schrödinger Glide with a binding region localized to the BAF. Pose of 2t16 from Figure **6B** included for comparison. Surface of exposed BAF shown in grey wireframe and clipped to aid in visualization.

(B) Residues from $BAX^{\Delta\alpha8}$:BIM simulated to interact with 2t16 or 2t16-NN-EtOH from **A** were compared and colored green or red, respectively, according to the number of poses and specificity for one of the ligands. Several determined poses are visualized to compare specificity and consistency of predicted binding.

(C) LUVs permeabilized by WT or BAF mutant BAX activated by BIM-BH3 (0.125–2 µM) to demonstrate no loss in activity resulting from mutations.

(D) Heatmap visualization of normalized endpoint LUV permeabilization data from **C**.

(E) Data summarized by Figure **6G**. LUVs permeabilized by WT or BAF mutant BAX activated by 2t16 (6.5–50 µM).

(F) Data summarized by Figure **6G**. As in **E** with BAX primed by BIM-BH3 peptide (0.15 µM).

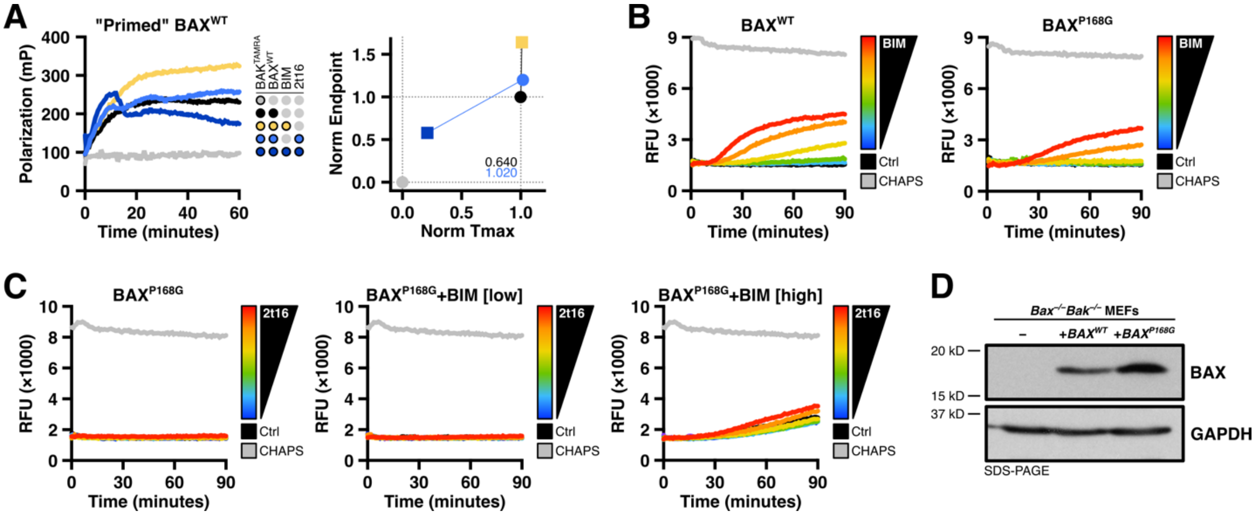

Supplementary Figure 9

Supplementary Figure 9: Mutating proline 168 disrupts 2t-hexadecenal synergy with BAX and BIM (Related to Figure 7)

(A) Left: BAX^{WT} (60 nM) was combined with a non-activating concentration of BIM-BH3 peptide (0.15 µM) and 2t16 (3 µM), followed by BAK^{TAMRA} (50 nM), and subjected to FLAMBE. Right: Parameterized FLAMBE data in the absence or presence of BIM-BH3 (circle and square datapoints, respectively). Annotations report the magnitude of shift between data with and without BIM-BH3. The parameterized trendline for BAX^{WT} is included for comparison in Figure **7D**.

(B) LUVs permeabilized by WT or P168G BAX activated by BIM-BH3 (0.125–2 µM).

(C) Data summarized by Figure **7F**. LUVs permeabilized by BAX^{P168G} (100 nM) treated with 2t16 (6.5–50 µM) ± BIM-BH3 peptide (0.5, 2 µM). Bliss synergy scores were determined from endpoint data and reported in Figure **7G**.

(D) Western blot confirming expression of BAX protein in transduced *Bax*$^{-/-}$*Bak*$^{-/-}$ MEFs.

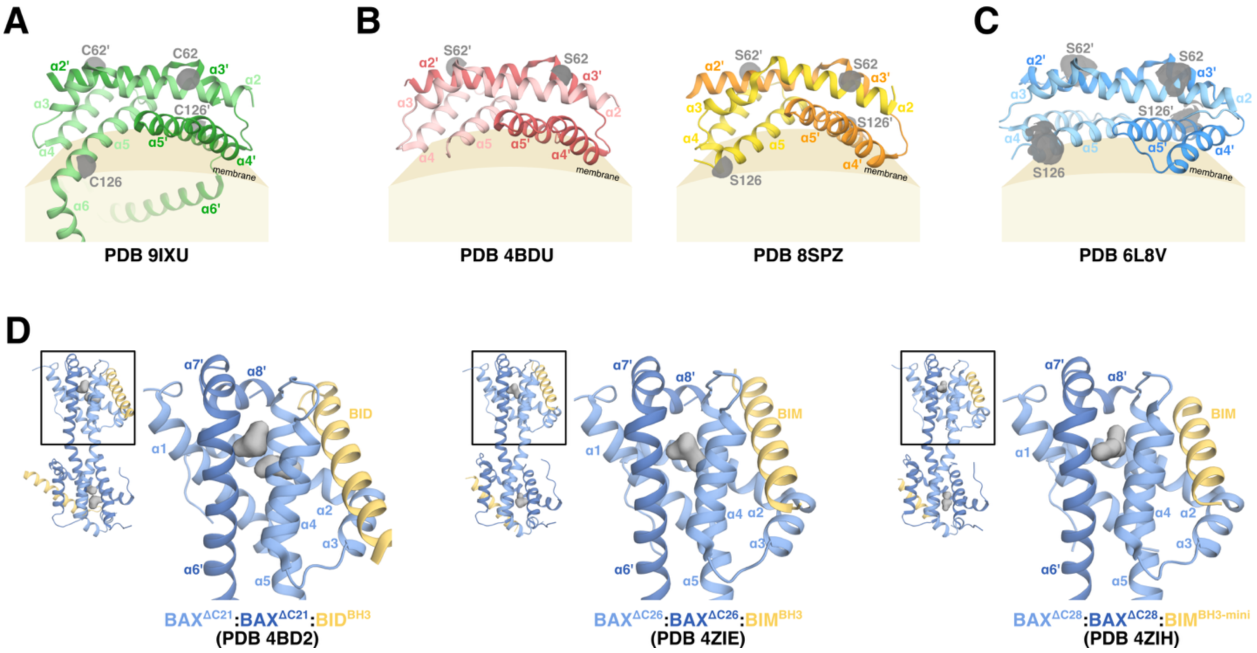

Supplementary Figure 10

Supplementary Figure 10: BAX structures provide insights into the orientation of reactive cysteines and the BAF during the BAX activation continuum

(A–C) Structures of active BAX BH3-in-groove dimers oriented with membranes. When included in the structure, the surfaces of cysteine (or substitution) sidechains are visualized to illustrate cytosol-facing or membrane-facing orientation. The orientation of each cysteine may explain how they were differentially targeted by prior studies assessing 2t-hexadecenal modification of BAX.

(A) Two chains from the oligomeric BAX structure with native C62 and C126 shown (PDB: 9IXU, chain A and chain B).

(B) Left: Two chains of dimeric BAX structure with mutated S62 shown; residue 126 is not included in structure (PDB: 4BDU, chain A and chain B). Right: Two chains of oligomeric BAX structure with mutated S62 and S126 shown (PDB: 8SPZ, chain A and chain B).

(C) NMR structure of BAX dimer (PDB: 6L8V, ensemble 1). Surface depiction of mutated S62 and S126 residues is an overlay representing the 20 state ensemble.

(D) Examples of domain-swapped core-latch dimer pairs bound to a variety of BH3 peptides each exhibiting a conserved cavity in the protein core. Cavity determinations and visualization were performed with PyMOL using a cavity radius of 2 and a cavity cutoff of -4.5.
