# Supplementary material for: A gated hydrophobic funnel within BAX binds bioactive lipids to potentiate pro-apoptotic function
Source: Nat Commun. 2026 Feb 25;17:3180. doi: 10.1038/s41467-026-69836-9 (PMC13046825; doi:10.1038/s41467-026-69836-9)
Supplement: Supplementary file 2 — Reporting Summary [file 41467_2026_69836_MOESM2_ESM.pdf]

nature portfolio

Corresponding author(s): Jerry Edward Chipuk

Last updated by author(s): Jan 14, 2026

Reporting Summary

Nature Portfolio wishes to improve the reproducibility of the work that we publish. This form provides structure for consistency and transparency in reporting. For further information on Nature Portfolio policies, see our Editorial Policies and the Editorial Policy Checklist.

Statistics

For all statistical analyses, confirm that the following items are present in the figure legend, table legend, main text, or Methods section.

n/a	Confirmed	
☐	☒	The exact sample size (*n*) for each experimental group/condition, given as a discrete number and unit of measurement
☐	☒	A statement on whether measurements were taken from distinct samples or whether the same sample was measured repeatedly
☐	☒	The statistical test(s) used AND whether they are one- or two-sided<br>*Only common tests should be described solely by name; describe more complex techniques in the Methods section.*
☒	☐	A description of all covariates tested
☐	☒	A description of any assumptions or corrections, such as tests of normality and adjustment for multiple comparisons
☐	☒	A full description of the statistical parameters including central tendency (e.g. means) or other basic estimates (e.g. regression coefficient) AND variation (e.g. standard deviation) or associated estimates of uncertainty (e.g. confidence intervals)
☐	☒	For null hypothesis testing, the test statistic (e.g. *F*, *t*, *r*) with confidence intervals, effect sizes, degrees of freedom and *P* value noted<br>*Give P values as exact values whenever suitable.*
☒	☐	For Bayesian analysis, information on the choice of priors and Markov chain Monte Carlo settings
☒	☐	For hierarchical and complex designs, identification of the appropriate level for tests and full reporting of outcomes
☒	☐	Estimates of effect sizes (e.g. Cohen's *d*, Pearson's *r*), indicating how they were calculated

Our web collection on statistics for biologists contains articles on many of the points above.

Software and code

Policy information about availability of computer code

Data collection	SPARKL data was collected and analyzed by either IncuCyte ZOOM software (v2018A) or Gen5 (v3.12) using a Cytation 7 (BioTek). Microplate-based fluorescence assays were measured using Gen5 (BioTek). MST data was analyzed using NanoTemper software. TOF mass spectrometry data was collected using Agilent MassHunter Bioconfirm (v12.0). Thermal shift data was collected using Applied Biosystems software. Unguided in silico docking was performed using SwissDock EADock (www.swissdock.ch); guided in silico docking was performed using Schrödinger Glide (v2023-1). Protein visualization and virtual mutagenesis screen was performed in PyMOL (v3.1). All necessary details are provided in the Methods section.
Data analysis	Curve fitting, non-linear regression, and statistical tests were accomplished using Graphpad Prism (v10). LC-MS data was analyzed using FragPip (v22.0), MSFragger, and PDV (https://github.com/wenbostar/PDV). HSQC NMR data was analyzed using TopSpin, CARA, and Microsoft Excel. All necessary details are provided in the Methods section.

For manuscripts utilizing custom algorithms or software that are central to the research but not yet described in published literature, software must be made available to editors and reviewers. We strongly encourage code deposition in a community repository (e.g. GitHub). See the Nature Portfolio guidelines for submitting code & software for further information.

Data

Policy information about availability of data

All manuscripts must include a data availability statement. This statement should provide the following information, where applicable:

- Accession codes, unique identifiers, or web links for publicly available datasets
- A description of any restrictions on data availability
- For clinical datasets or third party data, please ensure that the statement adheres to our policy

The data supporting the findings of this study are included in this published article (and its Supplementary Information files) and the source data underlying each figure are provided as a Source Data file. Materials generated as part of this study are available from the corresponding author upon request. Structures corresponding to PDB 1F16 [https://doi.org/10.2210/pdb1F16/pdb], 2K7W [https://doi.org/10.2210/pdb2K7W/pdb], and 5W60 [https://doi.org/10.2210/pdb5W60/pdb] were analyzed within this study; structures corresponding to PDB 4BD2 [https://doi.org/10.2210/pdb4BD2/pdb], 4BD6 [https://doi.org/10.2210/pdb4BD6/pdb], 4BDU [https://doi.org/10.2210/pdb4BDU/pdb], 4ZIE [https://doi.org/10.2210/pdb4ZIE/pdb], 6L8V [https://doi.org/10.2210/pdb6L8V/pdb], 8SPZ [https://doi.org/10.2210/pdb8SPZ/pdb], and 9IXU [https://doi.org/10.2210/pdb9IXU/pdb] were included as visual aids but were not used for investigation.

Research involving human participants, their data, or biological material

Policy information about studies with human participants or human data. See also policy information about sex, gender (identity/presentation), and sexual orientation and race, ethnicity and racism.

Reporting on sex and gender	Not applicable to this study.
Reporting on race, ethnicity, or other socially relevant groupings	Not applicable to this study.
Population characteristics	Not applicable to this study.
Recruitment	Not applicable to this study.
Ethics oversight	Not applicable to this study.

Note that full information on the approval of the study protocol must also be provided in the manuscript.

Field-specific reporting

Please select the one below that is the best fit for your research. If you are not sure, read the appropriate sections before making your selection.

☒ Life sciences ☐ Behavioural & social sciences ☐ Ecological, evolutionary & environmental sciences

For a reference copy of the document with all sections, see nature.com/documents/nr-reporting-summary-flat.pdf

Life sciences study design

All studies must disclose on these points even when the disclosure is negative.

Sample size	All experiments were performed with 2–4 technical replicates as appropriate, and were repeated and reproduced at least three times. Assays using only 2 replicates were not reported as average and deviation and no statistics were applied. Replicate experiments were conducted with materials from separate batches to confirm reproducibility (recombinant protein, liposomes, etc). Sample sizes were selected based on standard practices for these assays in the cell death field, as referenced in the manuscript, and provided reproducible results. Details of sample size are provided in figure legends and methods.
Data exclusions	In some assays, an individual technical replicate may have been omitted due to interference with data collection.
Replication	All experiments were replicated and reproduced at least three times. Whenever possible, separate reagents were utilized to ensure reproducibility (multiple protein purification batches, batches of liposomes, separate commercial reagents, etc.). When applicable, representative data are shown with statements regarding replicated experiments in the figure legends and methods.
Randomization	No experimental grouping or randomization was used in this study.
Blinding	Blinding is not applicable to the techniques utilized in this study.

Reporting for specific materials, systems and methods

We require information from authors about some types of materials, experimental systems and methods used in many studies. Here, indicate whether each material, system or method listed is relevant to your study. If you are not sure if a list item applies to your research, read the appropriate section before selecting a response.

Materials & experimental systems

n/a	Involved in the study	
☐	☒	Antibodies
☐	☒	Eukaryotic cell lines
☒	☐	Palaeontology and archaeology
☒	☐	Animals and other organisms
☒	☐	Clinical data
☒	☐	Dual use research of concern
☒	☐	Plants

Methods

n/a	Involved in the study	
☒	☐	ChIP-seq
☒	☐	Flow cytometry
☒	☐	MRI-based neuroimaging

Antibodies

Antibodies used	Antibodies: BAX, 1:500 dilution (Clone 2D2, Cat. No. sc-20067, Santa Cruz Biotechnology, Dallas, TX, USA); GAPDH, 1:1000 dilution (Clone 1E6D9, Cat. No. 60004, Proteintech, Rosemont, IL, USA); m-IgGk BP-HRP secondary antibody (Cat. No. sc-516102, Santa Cruz Biotechnology, Dallas, TX, USA).
Validation	All antibodies were validated by the manufacturer to interact with the proteins used in this study. Additionally, we validate target specificity through the use of knockout cell lines.

Eukaryotic cell lines

Policy information about cell lines and Sex and Gender in Research

Cell line source(s)	Bax+/+Bak+/+ and Bax−/−Bak−/− SV40-transformed MEFs were obtained from ATCC (Manassas, VA, USA); Bim−/−Bid−/− SV40-transformed MEFs were provided by Dr. Douglas Green (St. Jude Children's Research Hospital); Bax−/−Bak−/− double knockout MEFs reconstituted to express wild type or P168G BAX were provided by Dr. Evripidis Gavathiotis (Albert Einstein College of Medicine) and BAX expression was confirmed by western blot and GFP positivity from the pBabe IRES-GFP vector backbone.
Authentication	Cell lines were authenticated by the supplier, or by western to confirm knockout status of BCL-2 family proteins.
Mycoplasma contamination	All cell cultures were maintained in mycoplasm-free conditions as verified by the HEK-Blue Detection Kit (Cat. No. hb-det2, Invivogen, San Diego, CA, USA).
Commonly misidentified lines (See ICLAC register)	No commonly misidentified eukaryotic cell lines were used in this manuscript.

Plants

Seed stocks	Not applicable to this study.
Novel plant genotypes	Not applicable to this study.
Authentication	Not applicable to this study.
