# Supplementary figures and images for: A gated hydrophobic funnel within BAX binds bioactive lipids to potentiate pro-apoptotic function
Source: Nat Commun. 2026 Feb 25;17:3180. doi: 10.1038/s41467-026-69836-9 (PMC13046825; doi:10.1038/s41467-026-69836-9)

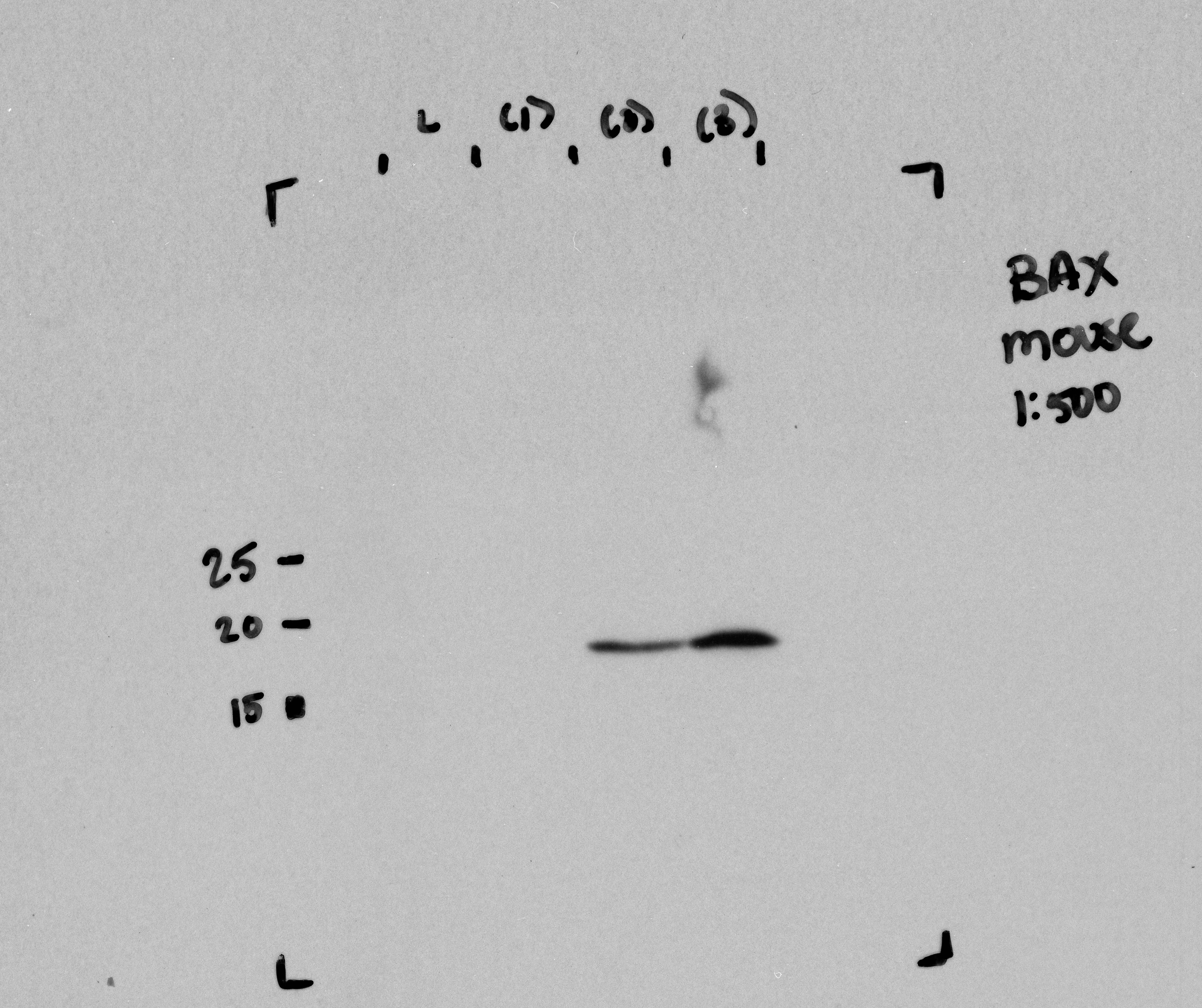

Supplement: Supplementary file 4 — Source Data [file 41467_2026_69836_MOESM4_ESM.zip › SourceDataFiles_v2/SourceData_FigS9D_BAX.jpg]

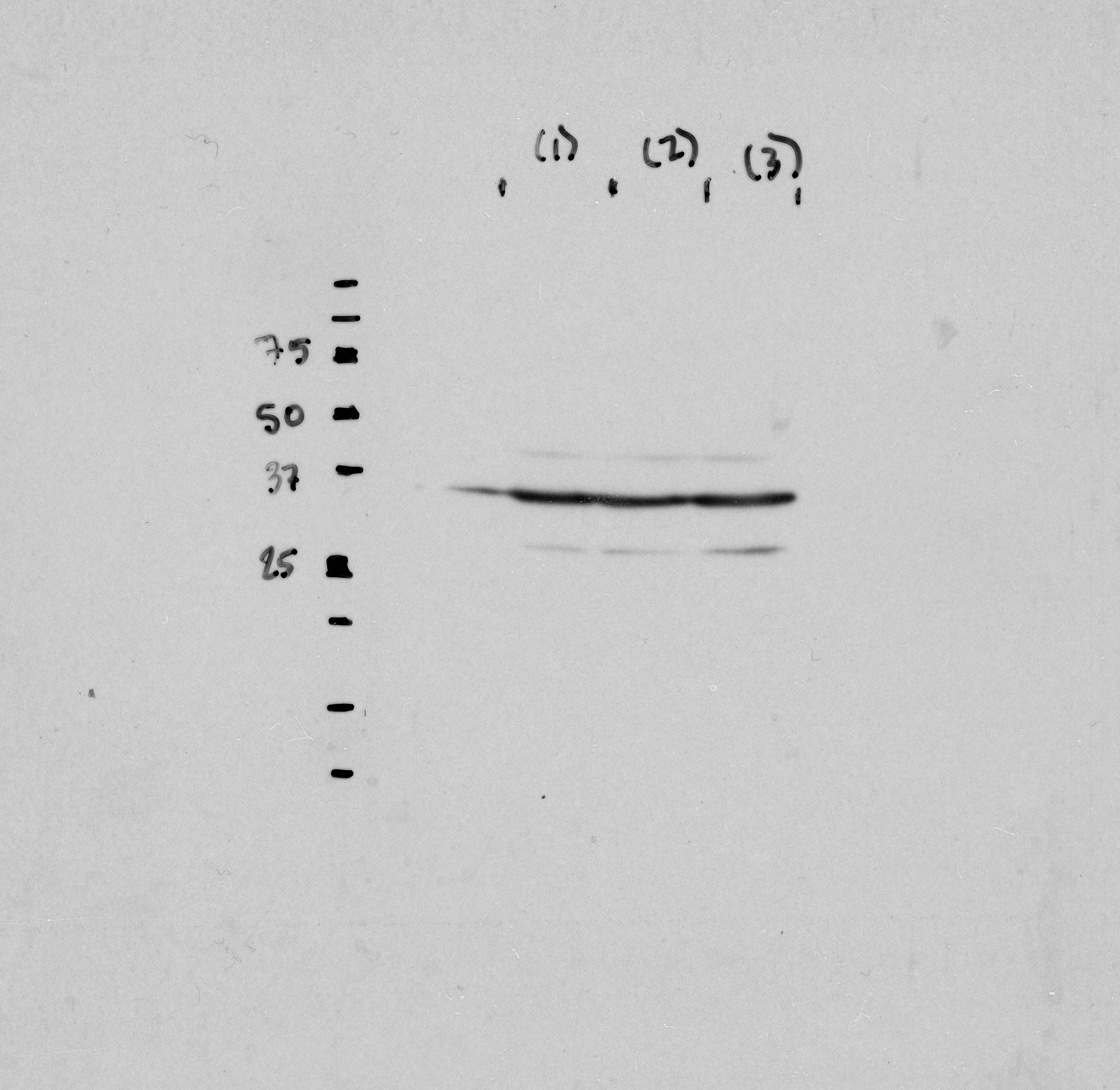

Supplement: Supplementary file 4 — Source Data [file 41467_2026_69836_MOESM4_ESM.zip › SourceDataFiles_v2/SourceData_FigS9D_GAPDH.jpg]
